# Supplementary material for: A PSA SNP associates with cellular function and clinical outcome in men with prostate cancer
Source: Nat Commun. 2024 Nov 6;15:9587. doi: 10.1038/s41467-024-52472-6 (PMC11541583; doi:10.1038/s41467-024-52472-6)
Supplement: Supplementary file 1 — Supplementary Information [file 41467_2024_52472_MOESM1_ESM.pdf]

Supplementary Information

Supplementary Table 1. Participant characteristics

Supplementary Table 2. Risk of prostate cancer for rs17632542 SNP

Supplementary Table 3: Frequency distribution for the rs17632542 SNP

Supplementary Table 4. Primers used for the study

Supplementary Figure 1. KLK3 expression in overexpression models

Supplementary Figure 2. Digital spheroid analysis of PC-3, LNCaP and MSK3 cells

Supplementary Figure 3. Confocal Microscopy for PSA and vector transfected PC3 cells on OBM constructs

Supplementary Figure 4. Effect of rs17632542 SNP on PC-3 cell metastasis in an experimental metastasis mouse model

Supplementary Figure 5. Proteolysis of peptide and full-length protein substrates by mature PSA protein variants

Supplementary Figure 6. Overall- and metastasis-free survival of MDC and VIP cohorts for the rs17632542 SNP

Supplementary Note 1. Information and acknowledgements for consortia contributing to the analysis and for individual study groups within the PRACTICAL Consortium.

References

Supplementary Table 1. Participant characteristics

Strata	PCa[a]		Family History Controls		Family History Cases		Risk - case only			Advanced[b]	
	controls	cases	No	Yes	No	Yes	High	Intermediate	Low	No	Yes
**PRACTICAL Consortium**											
Australia	1557	3996	798	114	768	618	1162	2102	118	169	132
Belgium	103	166	0	0	28	12	112	44	4	3	8
Bulgaria	89	192	89	0	192	0	108	77	6	0	0
Canada	455	668	338	77	486	155	96	314	143	0	0
Croatia	149	146	149	0	118	0	75	57	9	2	10
Denmark	1031	2057	0	0	766	87	802	1017	95	91	75
Finland	1183	2421	0	62	0	70	675	1021	543	79	148
France	691	923	606	85	629	294	417	501	3	8	13
Germany	493	781	484	9	578	203	308	424	7	64	33
Multi_Center	693	877	0	0	0	0	161	331	94	21	28
Nederland	65	71	0	0	0	0	0	0	0	0	0
Norway	0	1443	0	0	0	0	51	484	0	182	761
Poland	317	484	0	0	432	5	241	175	56	0	0
Portugal	180	374	0	0	181	190	182	161	29	0	1
Spain	819	1322	434	34	659	122	359	689	223	60	34
Sweden	2834	5976	750	79	1436	294	1599	2473	1380	539	624
UK	10854	17565	4881	1156	9129	3543	6396	5302	1566	1073	2307
USA	10488	10479	7410	1060	5977	1599	1303	3936	1766	1313	579
**Total**	**32001**	**49941**	**15939**	**2676**	**21379**	**7192**	**14047**	**19108**	**6042**	**3604**	**4753**

[a]Prostate Cancer; [b]Identification of advanced disease cases was based on Gleason score 8+, metastatic disease, PSA>100 or death from PCa.

Supplementary Table 2. Risk of prostate cancer for rs17632542 SNP

Analysis	OR (95% CI)[a]	P-value
**All prostate cancers**	0.70 (0.67-0.73)	9.6E-69
**Positive family history status**	0.75 (0.71-0.79)	2.7E-26
**Age of disease onset**	0.75 (0.71-0.79)	5.2E-29
**High risk[b] vs Low[c]**	1.58 (1.42-1.76)	1.23E-17
**High risk vs Low/Intermediate[d]**	1.42 (1.33-1.51)	1.41E-26
**Risk lethal vs controls**	1.33 (1.16-1.51)	2.29E-05

[a]Odds-ratio and (95% confidence interval); we defined [b]High risk as tumour stage T3/T4 or N1 or M1 or Gleason score ≥8 or PSA >20 ng/mL; [c]low risk as tumour stage ≤T1 and Gleason score ≤6 and PSA <10 ng/mL; and [d]intermediate risk as tumour stage T2 or Gleason score=7 or PSA=10-20 ng/mL. Association between the rs17632542 SNP and PCa risk was analysed using the per-allele trend test, adjusted for study relevant covariates using logistic regression and seven principal components derived from analysis of the whole iCOGS and OncoArray dataset. Odds Ratios and 95% confidence intervals were derived using SNPTEST or an in-house C++ program. Tests of homogeneity of the ORs across strata were calculated using a likelihood ratio test. In a case-only analyses, Cox proportional hazards regression was used to estimate associations of SNP.

Supplementary Table 3: Frequency distribution for the rs17632542 SNP

Category	Coding	TT	CT	CC	Total	Genotype Frequency		
						p(TT)	p(CT)	p(CC)
T-Stage	T0	19	4	0	23	0.826087	0.173913	0
	T1	13433	1408	38	14879	0.902816	0.09463	0.002554
	T2	13496	1543	45	15084	0.894723	0.102294	0.002983
	T3	6317	926	41	7284	0.867243	**0.127128**	0.005629
	T4	686	103	8	797	0.860728	**0.129235**	0.010038
Total		33951	3984	132	38067			
GLEASON score_Range	1	306	38	1	345	0.886957	0.110145	0.002899
	2	7697	890	32	8619	0.893027	0.10326	0.003713
	3	1182	203	8	1393	0.848528	**0.145729**	0.005743
	4	3	0	0	3	1	0	0
Total		9188	1131	41	10360			
N-Stage	N0	11883	1402	43	13328	0.891582	0.105192	0.003226
	N1	1001	182	12	1195	0.837657	**0.152301**	0.010042
Total		12884	1584	55	14523			
M-Stage	M0	13839	1709	55	15603	0.886945	0.10953	0.003525
	M1	1413	215	8	1636	0.863692	**0.131418**	0.00489
Total		15252	1924	63	17239			
SEER Staging	Local	27928	3056	85	31069	0.898902	0.098362	0.002736
	Regional	6454	948	43	7445	0.866891	**0.127334**	0.005776
	Distant	1421	216	8	1645	0.86383	**0.131307**	0.004863
Total		35803	4220	136	40159			

Significant frequencies for the genotype highlighted in bold.

Detailed description for each "Category"

Category	Variable name	Description	Coding
Cancer Stage	T-Stage	T-Stage: Size or direct extent of the primary tumour ('T')	T0=no evidence of tumour, T1=tumour present, but not detectable clinically or with imaging, T2=the tumour can be felt (palpated) on examination, but has not spread outside the prostate, T3=the tumour has spread through the prostatic capsule, T4=the tumour has invaded other nearby structures
Gleason	Gleason score_Range	Range based on Gleason score	1=Gleason score<5. 2=Gleason score 5,6,7. 3=Gleason score 8, 9, 10. 4=undifferentiated.
	N-Stage	Degree of spread to regional lymph nodes ('N')	N0=there has been no spread to the regional lymph nodes, N1=there has been spread to the regional lymph nodes
	M-Stage	Presence of metastasis ('M')	M0=there is no distant metastasis, M1=there is distant metastasis
	SEER Staging	Prostate cancer SEER staging	Local=Confined to the prostate, Regional=Direct extension involving adjacent local structures and local lymph node, Distant=Direct extension or metastasis

Supplementary Table 4. Primers used for the study

Gene	Primer sequences (5' to 3')	Reference/Source
KLK3 (Exon 2-3)	F1-AGTGCGAGAAGCATTCCCAAC R1-AACAAAAGCGTGATCTTGCTGG	[1]
GAPDH	F-GCAAATTCCATGGCACCGT R- TCGCCCCACTTGATTTTGG	[2]
pcDNA3.1 vector sequencing primers	F-TAATACGACTCACTATAGGG R-TAGAAGGCACAGTCGAGG	(Invitrogen™)
7SL	F-ATCGGGTGTCCGCACTAAGTT R-CAGCACGGGAGTTTTGACCT	(Life Technologies)
Alpha-signal	GGACGGATCCAAACGATGAGATTTCCTTCA	Sigma Aldrich, Australia
PSA mature	F-CTCTCGAGAAAAGAATTGTGGGAGGCTGGGAGT R-CAGCCTCCCACAATTCTTTTCTCGAGAGATACC	Sigma Aldrich, Australia
Site-directed mutagenesis primers for PSA-inactive mutant Ala^{195}	F-GCACCTGCTCGGGTGATGCTGGGGGCCCACTTGTC R-GACAAGTGGGCCCCCAGCATCACCCGAGCAGGTGC	(Primer X)
Site-directed mutagenesis primers for rs17632542	F-GTGGACCTCCATGTTACTTCCAATGACGTGTG R-CACACGTCATTGGAAGTAACATGGAGGTCCAC	(Primer X)

Supplementary Figure 1

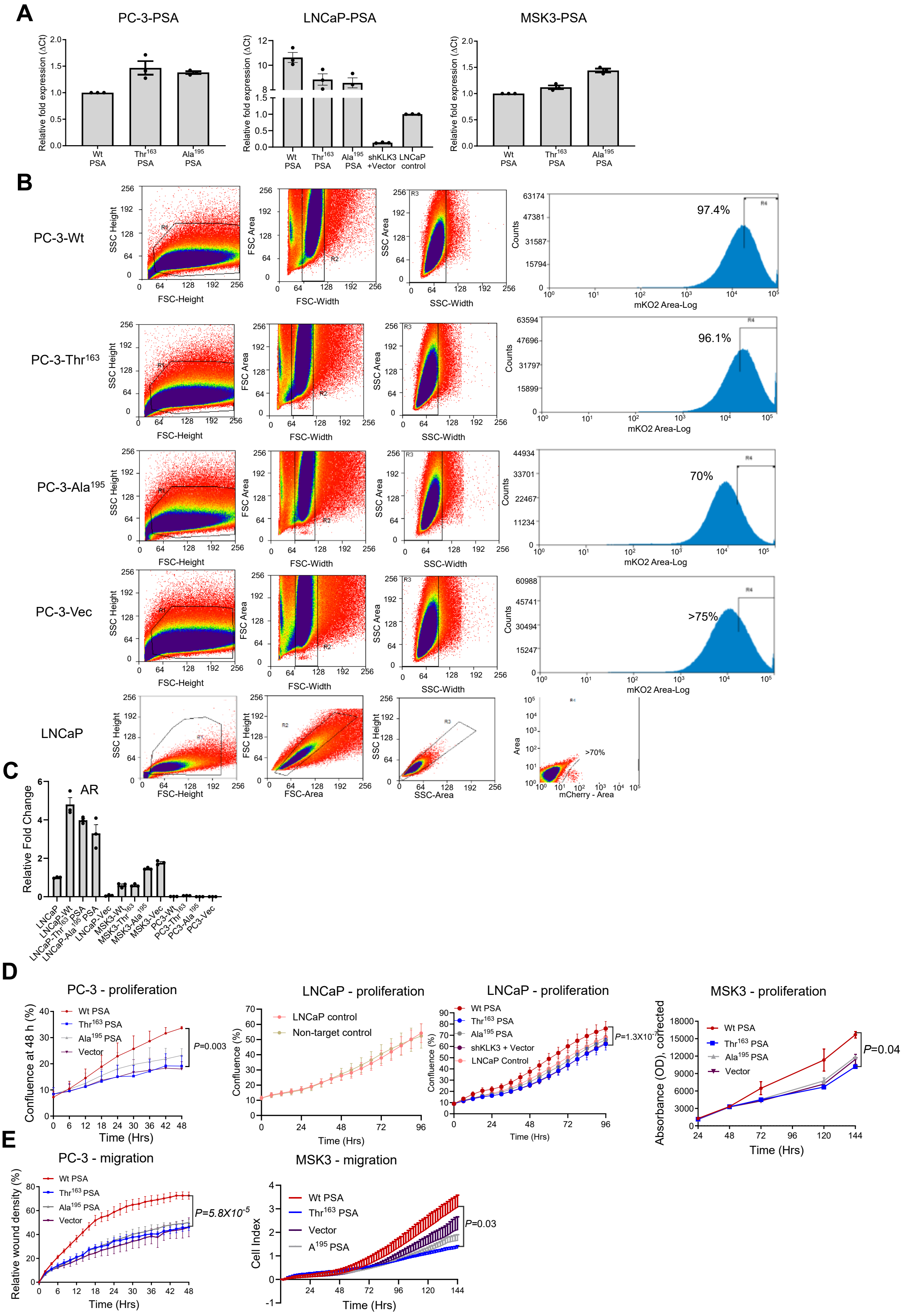

Supplementary Figure 1. *KLK3* expression in overexpression models.

A) Representative mRNA analysis demonstrating the expression of PSA in PSA transfected PC-3, LNCaP and MSK3 clones: Wt PSA, Thr^{163} PSA and inactive mutant Ala^{195} PSA. For LNCaP cells, PSA expression in vector (PSA knock-down (shKLK3) and transfection with vector pLEX307-GFP) and control non-transfected LNCaP cells are shown (*n*=3 independent experiments). **B)** FACS analysis showing mKO2 positive cells in PC-3 cells and mCherry positive cells in LNCaP cells. The % of transfection efficiency is indicated. **C)** *AR* expression in LNCaP (non-transfected) and PSA transfected PC-3, LNCaP and MSK3 clones: Wt PSA, Thr^{163} PSA and inactive mutant Ala^{195} PSA (*n*=2 independent experiments). **C)** Proliferation rate (confluence %) monitored in the IncuCyte live cell imaging system for PC-3, LNCaP and MSK3 cells expressing PSA variants, vector control and non-target control (LNCaP) (*n*=3 independent experiments). **D)** Migration rate (relative wound density %) measured by IncuCyte live cell imaging system for PC-3 cells and migration (Cell index) for MSK3 cells measured using the xCELLigence system, expressing PSA variants and vector control (*n*=3 independent experiments). All error bars represent mean ± SEM. Statistical analyses were determined by one-way ANOVA followed by Dunn's multiple comparison test (D, E). Source data are provided as a Source Data file.

Supplementary Figure 2

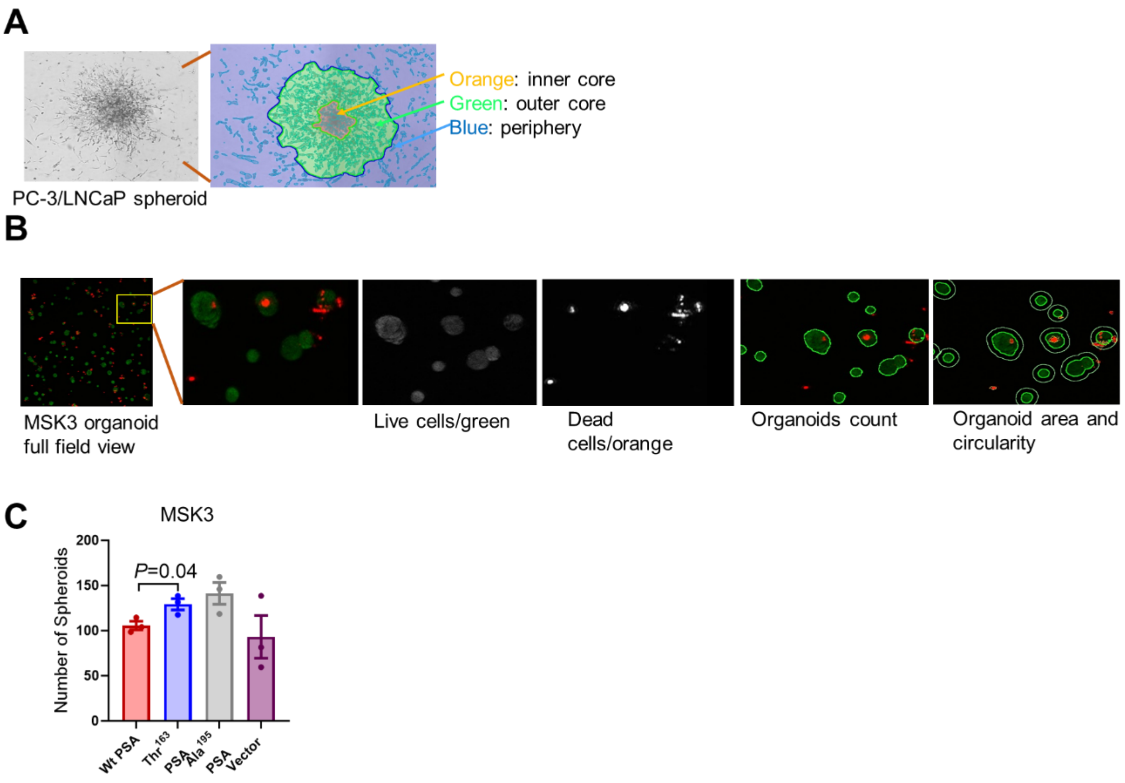

Supplementary Figure 2. Digital spheroid analysis of PC-3, LNCaP and MSK3 cells.

A) A gray channel image generated from the original image was corrected to reduce the background. A density image was generated for the detection of spheroids as cell agglomerations with high cell numbers per area and their separation from isolated cells distributed across the wells. Positive objects (confirmed spheroids) were split into three areas, with the green contour indicating the outer core, the orange contour labelling the inner core and the blue contour highlighting the regions with detectable cells in the periphery. Quantitative analyses for the area and circularity of PC-3 or LNCaP spheroids were determined by the StrataQuest^{TM} software. **B)** Dead (red) and live (green) cells count and mean intensity within the spheroid was detected based on setting thresholds, spheroid number, and event area for spheroid area were measured for the MSK3 spheroids. **C)** Number of MSK3 spheroids in selected field (average number from two images; *n*=3 independent experiments). See also Figure 2A–J. All error bars represent mean ± SEM. Statistical analyses were determined by two-sided Unpaired *t* test with Welch's correction. Source data are provided as a Source Data file.

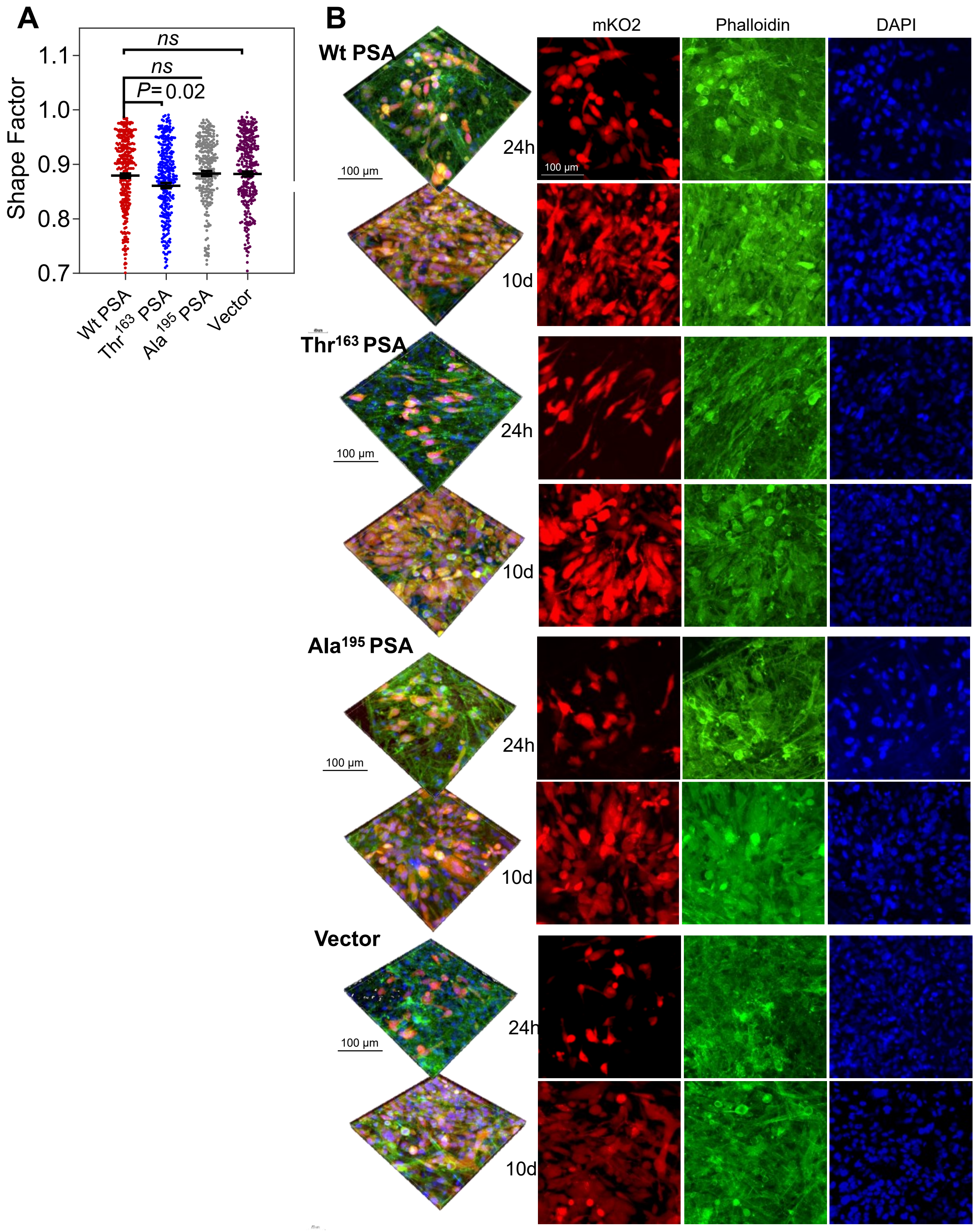

Supplementary Figure 3. Confocal Microscopy for PSA and vector transfected PC-3 cells on OBM constructs.

A) Shape Factor of PC-3 cells to OBM constructs after 12 h co-culture. **B)** Confocal laser microscopy images from PC-3/OBM constructs after 1 day and 10 days co-culture showing, from left to right, a volume snapshot of all channels and the maximum projections of z-stacks (mKO2 (red) for PC-3, GFP (green) for Phalloidin, and DAPI channel (blue) showing nuclei of

both cancer cells and osteoblasts. For **A-B**, 2 technical replicates were used, 4-5 fields of view/replicate, for a total of 120-230 cells per condition. *P* values on all groups were evaluated by one-way ANOVA followed by Games-Howell post hoc analysis. See also Figure 2H-J. Source data are provided as a Source Data file.

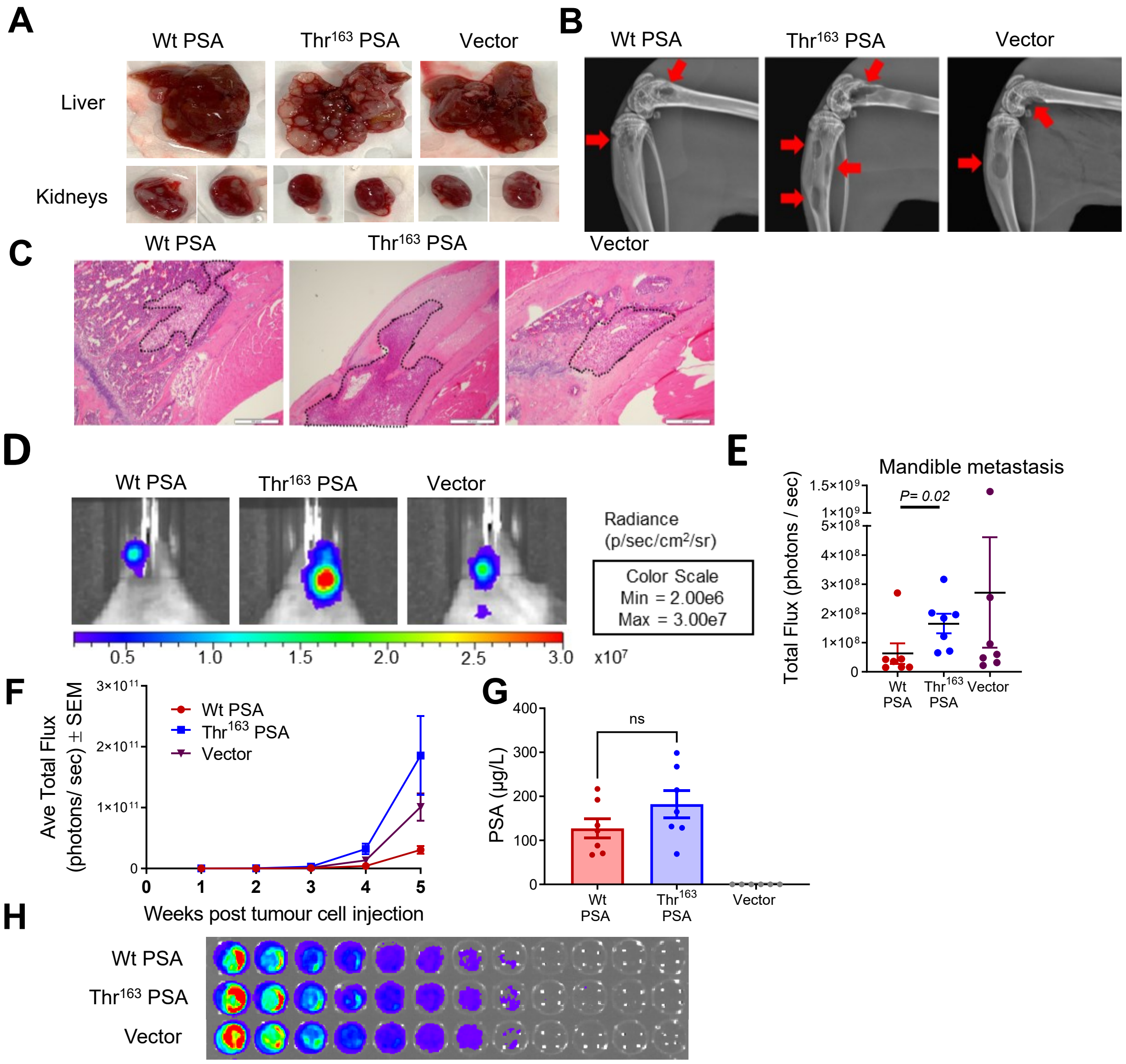

Supplementary Figure 4. Effect of rs17632542 SNP on PC-3 cell metastasis in an experimental metastasis mouse model.

A) Representative photographs of resected liver and kidneys from mice following cardiac injection of PC-3-Wt/Thr^{163} PSA (*n*=7 mice/group). Increased tumour lesions are observed in the livers of Thr^{163} PSA injected mice. **B)** X-ray images of tumour-bearing hind legs of mice; red areas indicate areas of bone degradation, suggesting presence of tumour. **C**) H&E staining of tumour-bearing hind leg bones. **D**) Representative bioluminescence images of tumour-bearing mandibles of mice (week 4) post cardiac inoculation. **E**) Scatter plots of tumour bioluminescence based on region of interest (ROI) drawn over the jaw; horizontal line indicates median value. Statistical analysis was Dunn's multiple comparisons test. **F**) Mean bioluminescence values from ROI drawn over entire animals from each group, over multiple weeks. **G**) Serum concentration of total PSA at endpoint from mice injected intracardiac with tumour cells. All error bars represent mean ± SEM. Statistical analyses were determined by two-sided Student's *t* test (*n*=7 mice/group). **H**) *In-vitro* bioluminescence images of cell lines seeded by 2-fold serial dilution, starting at 50,000 cells per well. Also see Figure 2N-O.

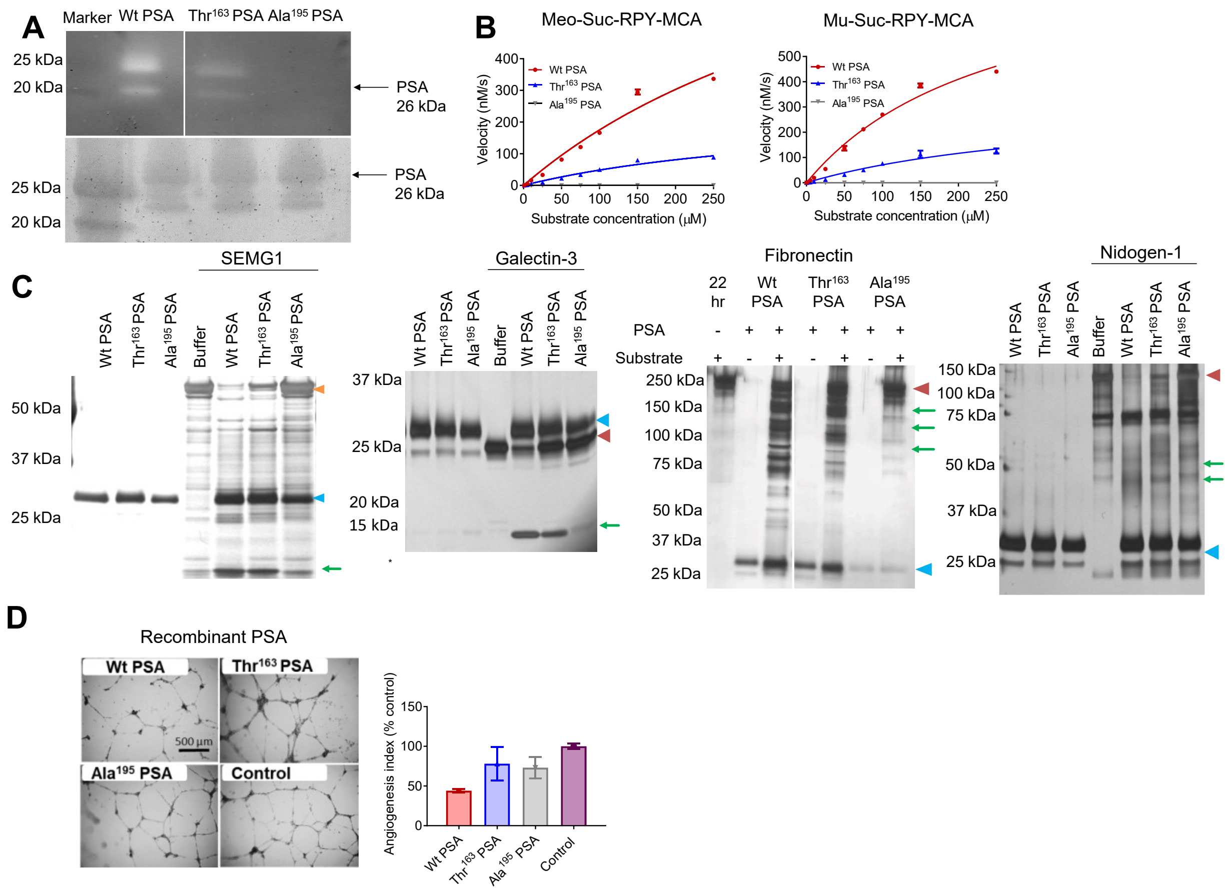

Supplementary Figure 5. Proteolysis of peptide and full-length protein substrates by mature PSA protein variants.

A) Casein zymography of Wt PSA and Thr^{163} PSA: One µg of Wt PSA, Thr^{163} PSA and inactive mutant Ala^{195} PSA (from left to right) were resolved on a 10% casein zymogram Protein Gel (Invitrogen) followed by Coomassie brilliant blue R-250 (0.25% w/v) staining. Clear zones due to protease activity were observed in the Wt PSA and Thr^{163} PSA lanes only. The bottom gel represents the silver stain analysis to indicate equal protein loaded into the wells. **B)** Michaelis-Menten kinetics for PSA protein variants: Michaelis-Menten kinetic analysis of Wt PSA (red), Thr^{163} PSA (blue) and inactive mutant Ala^{195} PSA (grey) for two substrates MeO-Suc-RPY-AMC and Mu-HSSKLQ-MCA. Kcat values showed the Thr^{163} PSA protein variant had decreased substrate activity in comparison to Wt PSA (mean ± SEM; *n*=3 independent experiments). Also see legend to Figure 3B. (**C**) Silver stain analysis of mature PSA variants (0.2 µM) incubated for 22 h with full-length substrates (semenogelin-1, galectin-3, fibronectin, nidogen-1, and laminin α-4) (0.5 µM) at 37°C, indicated that the Thr^{163} PSA isoform exhibited lower proteolytic activity compared to the wild type (Wt) PSA. Ala^{195} PSA had less effect. Wt PSA efficiently cleaved full-length fibronectin and laminin α-4, while partial proteolysis was observed with nidogen-1. The full-length proteins (orange arrow), PSA band (blue arrow) and their corresponding molecular weights are indicated. Cleaved products of the substrates (green arrows) due to PSA proteolytic activity are indicated to the right. High molecular weight bands that may correspond to the dimers of the full-length protein or their aggregates were observed above their expected size bands. Molecular weight of the protein standard (kDa) is indicated to the left. Also see Figure 3C-D. **D)** HUVECs treated with different recombinant PSA

protein variants (250 nM) (Wt, Thr^{163} and Ala^{195} PSA) and the graph to the right represents the angiogenesis index. Thr^{163} PSA exhibited lower anti-angiogenic potential compared to Wt PSA (*n*=2, mean ± SEM). Scale bar is 500 μm. Also see Figure 3F.

Supplementary Figure 6

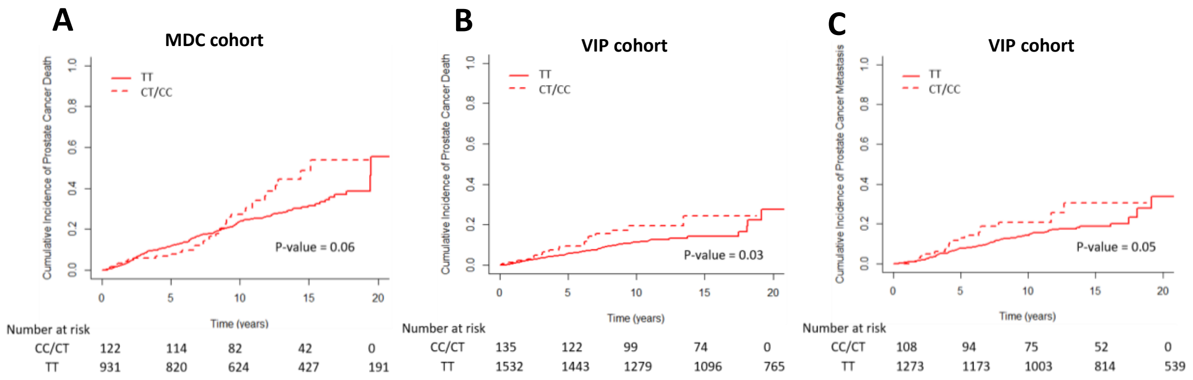

Supplementary Figure 6. Overall- and metastasis-free survival of MDC and VIP cohorts for the rs17632542 SNP.

A-B) Overall survival as measured by cumulative incidence of death from PCa for the rs17632542 SNP in **A)** MDC (*n*=1,053), HR= 1.39, 95% CI=0.98-1.98, *P*=0.06; and **B)** VIP cohorts (*n*=1,644), HR=1.69, 95% CI=1.07-2.65, *P*=0.03. **(C)** Metastasis free survival analysis estimated by Kaplan Maier plot in the VIP cohort of 1,381 prostate cancer cases. rs17632542 is associated with metastasis-free survival time in VIP cohort (HR=1.65, 95% CI=1.03-2.62, P=0.05).

Supplementary Note 1. Information and acknowledgements for consortia contributing to the analysis and for individual study groups within the PRACTICAL Consortium.

Funding Acknowledgements

This work was supported by the Canadian Institutes of Health Research, European Commission's Seventh Framework Programme grant agreement n° 223175 (HEALTH-F2-2009-223175), Cancer Research UK Grants C5047/A7357, C1287/A10118, C1287/A16563, C5047/A3354, C5047/A10692, C16913/A6135, and The National Institute of Health (NIH) Cancer Post-Cancer GWAS initiative grant: No. 1 U19 CA 148537-01 (the GAME-ON initiative).

We would also like to thank the following for funding support: The Institute of Cancer Research and The Everyman Campaign, The Prostate Cancer Research Foundation, Prostate Research Campaign UK (now PCUK), The Orchid Cancer Appeal, Rosetrees Trust, The National Cancer Research Network UK, The National Cancer Research Institute (NCRI) UK. We are grateful for support of NIHR funding to the NIHR Biomedical Research Centre at The Institute of Cancer Research, The Royal Marsden NHS Foundation Trust, and Manchester NIHR Biomedical Research Centre. The Prostate Cancer Program of Cancer Council Victoria also acknowledge grant support from The National Health and Medical Research Council, Australia (126402, 209057, 251533, 396414, 450104, 504700, 504702, 504715, 623204, 940394, 614296,), VicHealth, Cancer Council Victoria, The Prostate Cancer Foundation of Australia, The Whitten Foundation, PricewaterhouseCoopers, and Tattersall's. EAO, DMK, and EMK acknowledge the Intramural Program of the National Human Genome Research Institute for their support.

Genotyping of the OncoArray was funded by the US National Institutes of Health (NIH) [U19 CA 148537 for ELucidating Loci Involved in Prostate cancer SuscEptibility (ELLIPSE) project and X01HG007492 to the Center for Inherited Disease Research (CIDR) under contract number HHSN268201200008I]. Additional analytic support was provided by NIH NCI U01 CA188392 (PI: Schumacher).

Research reported in this publication also received support from the National Cancer Institute of the National Institutes of Health under Award Numbers U10 CA37429 (CD Blanke), and UM1 CA182883 (CM Tangen/IM Thompson). The content is solely the responsibility of the authors and does not necessarily represent the official views of the National Institutes of Health.

Funding for the iCOGS infrastructure came from: the European Community's Seventh Framework Programme under grant agreement n° 223175 (HEALTH-F2-2009-223175) (COGS), Cancer Research UK (C1287/A10118, C1287/A 10710, C12292/A11174, C1281/A12014, C5047/A8384, C5047/A15007, C5047/A10692, C8197/A16565), the National Institutes of Health (CA128978) and Post-Cancer GWAS initiative (1U19 CA148537, 1U19 CA148065 and 1U19 CA148112 - the GAME-ON initiative), the Department of Defence (W81XWH-10-1-0341), the Canadian Institutes of Health Research (CIHR) for the CIHR Team in Familial Risks of Breast Cancer, Komen Foundation for the Cure, the Breast Cancer Research Foundation, and the Ovarian Cancer Research Fund.

Additional funding and acknowledgments from studies in PRACTICAL:

Aarhus
This study was supported by Innovation Fund Denmark, the Danish Cancer Society and The Velux Foundation (Veluxfonden).

The Danish Cancer Biobank (DCB) is acknowledged for biological material.

AHS
This work was supported by the Intramural Research Program of the NIH, National Cancer Institute, Division of Cancer Epidemiology and Genetics (Z01CP010119).

COSM
COSM is funded by The Swedish Research Council (grant for the Swedish Infrastructure for Medical Population-based Life-course Environmental Research – SIMPLER), the Swedish Cancer Foundation.

CPCS1/CPCS2
Department of Clinical Biochemistry, Herlev and Gentofte Hospital, Copenhagen University Hospital, Herlev Ringvej 75, DK-2730 Herlev, Denmark
We thank participants and staff of the Copenhagen General Population Study for their important contributions.

EPIC
The coordination of EPIC was financially supported by the European Commission (DG-SANCO) and the International Agency for Research on Cancer. The national cohorts (that recruited male participants) are supported by Danish Cancer Society (Denmark); German Cancer Aid, German Cancer Research Center (DKFZ), Federal Ministry of Education and Research (BMBF), Deutsche Krebshilfe, Deutsches Krebsforschungszentrum and Federal Ministry of Education and Research (Germany); the Hellenic Health Foundation (Greece); Associazione Italiana per la Ricerca sul Cancro-AIRC-Italy and National Research Council (Italy); Dutch Ministry of Public Health, Welfare and Sports (VWS), Netherlands Cancer Registry (NKR), LK Research Funds, Dutch Prevention Funds, Dutch ZON (Zorg Onderzoek Nederland), World Cancer Research Fund (WCRF), Statistics Netherlands (The Netherlands); Health Research Fund (FIS), PI13/00061 to Granada; , PI13/01162 to EPIC-Murcia), Regional Governments of Andalucía, Asturias, Basque Country, Murcia and Navarra, ISCIII RETIC (RD06/0020) (Spain); Swedish Cancer Society, Swedish Research Council and County Councils of Skåne and Västerbotten (Sweden); Cancer Research UK (14136 to EPIC-Norfolk; C8221/A29017 to EPIC-Oxford), Medical Research Council
(1000143 to EPIC-Norfolk, MR/M012190/1 to EPIC-Oxford) (United Kingdom). For information on how to submit an application for gaining access to EPIC data and/or biospecimens, please follow the instructions at http://epic.iarc.fr/access/index.php.

ESTHER
The ESTHER study was supported by a grant from the Baden Württemberg Ministry of Science, Research and Arts.
The ESTHER group would like to thank Hartwig Ziegler, Sonja Wolf, Volker Hermann, Heiko Müller, Karina Dieffenbach, Katja Butterbach for valuable contributions to the study.

FHCRC
The FHCRC studies were supported by grants R01-CA080122, R01-CA056678, R01-CA082664, and R01-CA092579, and K05-CA175147 from the US National Cancer Institute, National Institutes of Health, with additional support from the Fred Hutchinson Cancer Research Center (P30-CA015704).
We thank all the individuals who participated in these studies.

HPFS
The Health Professionals Follow-up Study was supported by grants UM1CA167552, CA133891, CA141298, and P01CA055075.

We are grateful to the participants and staff of the Physicians' Health Study and Health Professionals Follow-Up Study for their valuable contributions, as well as the following state cancer registries for their help: AL, AZ, AR, CA, CO, CT, DE, FL, GA, ID, IL, IN, IA, KY, LA, ME, MD, MA, MI, NE, NH, NJ, NY, NC, ND, OH, OK, OR, PA, RI, SC, TN, TX, VA, WA, WY.
ICGC

IMPACT
The IMPACT study was funded by The Ronald and Rita McAulay Foundation, CR-UK Project grant (Grant references (C5047/A21332, C5047/A13232 and C5047/A17528), The BRCA Research and Cure Alliance, Cancer Australia, AICR Netherlands A10-0227, Cancer Council Tasmania and Cancer Australia, grant number 1006349 (2011-2013), Prostate Cancer Foundation of Australia, grant number PCFA PRO4 (2008) and Cancer Councils of Victoria and South Australia, grant number 400048 (2006-2008), The Victorian Cancer Agency Clinical Trial Capacity CTCB08_14, Cancer Australia & Prostate Cancer Foundation of Australia (2014-2016) grant number 1059423, and Translational grants EOI09_50. , NIHR, EU Framework 6, Cancer Councils of Victoria and South Australia, Philanthropic donation to Northshore University Health System. We acknowledge funding from the Slovenian Research Agency, Research programme P3-0352. We thank CERCA Program / Generalitat de Catalunya for their institutional support. We acknowledge the support of the Asociación Española Contra el Cáncer (AECC), the Instituto de Salud Carlos III (organismo adscrito al Ministerio de Economía y Competitividad) and "Fondo Europeo de Desarrollo Regional (FEDER), una manera de hacer Europa" (PI10/01422, PI13/00285, PIE13/00022, PI16/00563, JR18/00011 and CIBERONC) and the Institut Català de la Salut and Autonomous Government of Catalonia (2009SGR290, 2014SGR338 and PERIS Project MedPerCan).
We acknowledge support from the National Institute for Health and Care Research (NIHR) to the Biomedical Research Centre at The Institute of Cancer Research and Royal Marsden Foundation NHS Trust, at Manchester University Foundation Trust (IS-BRC-1215-20007), the Oxford Biomedical Research Centre Program and the Cambridge Clinical Research Centre, NIHR Cambridge Biomedical Research Centre. We acknowledge the IMPACT study steering committee, collaborators and participants.

KULEUVEN
F.C. and S.J. are holders of grants from FWO Vlaanderen (G.0684.12N and G.0830.13N), the Belgian federal government (National Cancer Plan KPC_29_023), and a Concerted Research Action of the KU Leuven (GOA/15/017). TVDB is holder of a doctoral fellowship of the FWO.

MCC-Spain
"The study was partially funded by the ""Accion Transversal del Cancer"", approved on the Spanish Ministry Council on the 11th October 2007, by the Instituto de Salud Carlos III-FEDER (PI08/1770, PI09/00773-Cantabria, PI11/01889-FEDER, PI12/00265, PI12/01270, PI12/00715, PI15/00069), by the Fundación Marqués de Valdecilla (API 10/09), by the Spanish Association Against Cancer (AECC) Scientific Foundation and by the Catalan Government DURSI grant 2009SGR1489. Samples: Biological samples were stored at the Parc de Salut MAR Biobank (MARBiobanc; Barcelona) which is supported by Instituto de Salud Carlos III FEDER (RD09/0076/00036). Also sample collection was supported by the Xarxa de Bancs de Tumors de Catalunya sponsored by Pla Director d'Oncologia de Catalunya (XBTC). ISGlobal acknowledges support from the Spanish Ministry of Science and Innovation through the "Centro de Excelencia Severo Ochoa 2019-2023" Program (CEX2018-000806-S), and support from the Generalitat de Catalunya through the CERCA Program."
We acknowledge the contribution from Esther Gracia-Lavedan in preparing the data. We thank all the subjects who participated in the study and all MCC-Spain collaborators.

MCCS
"The Melbourne Collaborative Cohort Study (MCCS) cohort recruitment was funded by VicHealth and Cancer Council Victoria. The MCCS was further supported by Australian

National Health and Medical Research Council grants 209057 and 396414 and by infrastructure provided by Cancer Council Victoria. Cases and their vital status were ascertained through the Victorian Cancer Registry and the Australian Institute of Health and Welfare, including the National Death Index and the Australian Cancer Database.
The Prostate Cancer Program of Cancer Council Victoria also acknowledge grant support from The National Health and Medical Research Council, Australia (126402, 209057, 251533, 396414, 450104, 504700, 504702, 504715, 623204, 940394, 614296,), VicHealth, Cancer Council Victoria, The Prostate Cancer Foundation of Australia, The Whitten Foundation, PricewaterhouseCoopers, and Tattersall's."

MEC
The MEC was supported by NIH grants CA063464, CA054281, CA098758, and CA164973.

Oslo
CONOR was supported by grants from the Nordic Cancer Union, the Swedish Cancer Society (2012/823) and the Swedish Research Council (2014/2269).
The authors wish to acknowledge the services of CONOR, the contributing research centres delivering data to CONOR, and all the study participants.

PHS
The Physicians' Health Study was supported by grants CA34944, CA40360, CA097193, HL26490 and HL34595.
We are grateful to the participants and staff of the Physicians' Health Study and Health Professionals Follow-Up Study for their valuable contributions, as well as the following state cancer registries for their help: AL, AZ, AR, CA, CO, CT, DE, FL, GA, ID, IL, IN, IA, KY, LA, ME, MD, MA, MI, NE, NH, NJ, NY, NC, ND, OH, OK, OR, PA, RI, SC, TN, TX, VA, WA, WY.
PLCO
This PLCO study was supported by the Intramural Research Program of the Division of Cancer Epidemiology and Genetics, National Cancer Institute, NIH
The authors thank Drs. Christine Berg and Philip Prorok, Division of Cancer Prevention at the National Cancer Institute, the screening center investigators and staff of the PLCO Cancer Screening Trial for their contributions to the PLCO Cancer Screening Trial. We thank Mr. Thomas Riley, Mr. Craig Williams, Mr. Matthew Moore, and Ms. Shannon Merkle at Information Management Services, Inc., for their management of the data and Ms. Barbara O'Brien and staff at Westat, Inc. for their contributions to the PLCO Cancer Screening Trial. We also thank the PLCO study participants for their contributions to making this study possible.

PRAGGA
PRAGGA was supported by Programa Grupos Emergentes, Cancer Genetics Unit, CHUVI Vigo Hospital, Instituto de Salud Carlos III, Spain.
PRAGGA wishes to thank Victor Muñoz Garzón, Manuel Enguix Castelo, Sara Miranda Ponte, Carmen M Redondo, Manuel Calaza, Francisco Gude Sampedro, Joaquín González-Carreró and the staff of the Department of Pathology and Biobank of University Hospital Complex of Vigo, Instituto de Investigación Sanitaria Galicia Sur (IISGS), SERGAS, Vigo, Spain; Máximo Fraga, José Antúnez and the Biobank of University Hospital Complex of Santiago, Santiago de Compostela, Spain; and Maria Torres, Angel Carracedo and the Galician Foundation of Genomic Medicine.

PROCAP
PROCAP was supported by the Swedish Cancer Foundation (08-708, 09-0677).
We thank and acknowledge all of the participants in the PROCAP study. We thank Carin Cavalli-Björkman and Ami Rönnberg Karlsson for their dedicated work in the collection of data. Michael Broms is acknowledged for his skilful work with the databases. KI Biobank is acknowledged for handling the samples and for DNA extraction. We acknowledge The NPCR

steering group: Pär Stattin (chair), Anders Widmark, Stefan Karlsson, Magnus Törnblom, Jan Adolfsson, Anna Bill-Axelson, Ove Andrén, David Robinson, Bill Pettersson, Jonas Hugosson, Jan-Erik Damber, Ola Bratt, Göran Ahlgren, Lars Egevad, and Roy Ehrnström.

PROFILE
We would like to acknowledge the support of the Ronald and Rita McAulay Foundation, Cancer Research UK and Prostate Cancer UK/Movember Centre of Excellence. We also acknowledge support from the National Institute for Health and Care Research (NIHR) to the Biomedical Research Centre at The Institute of Cancer Research and Royal Marsden NHS Foundation Trust as well as the NIHR Programme Grant for Applied Research.
We acknowledge the PROFILE study steering committee and participants.

PROGReSS
This research was supported by Spanish Instituto de Salud Carlos III (ISCIII) funding, an initiative of the Spanish Ministry of Economy and Innovation partially supported by European Regional Development FEDER Funds (INT15/00070, INT16/00154, INT17/00133; PI19/01424; PI16/00046; PI13/02030; PI10/00164), and through the Autonomous Government of Galicia (Consolidation and structuring program: IN607B) given to A.Vega.
We would like to thank the patients for their contribution to the study

ProMPT
CRUK, NIHR, MRC, Cambridge Biomedical Research Centre
"ProtecT would like to acknowledge the support of The University of Cambridge, Cancer Research UK. Cancer Research UK grants [C8197/A10123] and [C8197/A10865] supported the genotyping team. We would also like to acknowledge the support of the National Institute for Health Research which funds the Cambridge Bio-medical Research Centre, Cambridge, UK. We would also like to acknowledge the support of the National Cancer Research Prostate Cancer: Mechanisms of Progression and Treatment (PROMPT) collaborative (grant code G0500966/75466) which has funded tissue and urine collections in Cambridge. We are grateful to staff at the Welcome Trust Clinical Research Facility, Addenbrooke's Clinical Research Centre, Cambridge, UK for their help in conducting the ProtecT study. We also acknowledge the support of the NIHR Cambridge Biomedical Research Centre, the NIHR HTA (ProtecT grant) and the NCRI / MRC (ProMPT grant) for help with the bio-repository. The UK Department of Health funded the ProtecT study through the NIHR Health Technology Assessment Programme (projects 96/20/06, 96/20/99). The ProtecT trial and its linked ProMPT and CAP (The Cluster Randomized Trial of PSA Testing for Prostate Cancer) studies are supported by Department of Health, England; Cancer Research UK grant number C522/A8649, C11043/A4286, C18281/A8145, C18281/A11326, and C18281/A15064, Medical Research Council of England grant number G0500966, ID 75466 and The NCRI, UK. The epidemiological data for ProtecT were generated though funding from the Southwest National Health Service Research and Development. DNA extraction in ProtecT was supported by USA Dept of Defense award W81XWH-04-1-0280, Yorkshire Cancer Research and Cancer Research UK. The authors would like to acknowledge the contribution of all members of the ProtecT study research group.

The views and opinions expressed therein are those of the authors and do not necessarily reflect those of the Department of Health of England. The bio-repository from ProtecT is supported by the NCRI (ProMPT) Prostate Cancer Collaborative and the Cambridge BMRC grant from NIHR.

We acknowledge support from the National Cancer Research Institute (National Institute of Health Research (NIHR) Collaborative Study: "Prostate Cancer: Mechanisms of Progression and Treatment (PROMPT)" (grant G0500966/75466).

We thank the National Institute for Health Research, Hutchison Whampoa Limited, the Human Research Tissue Bank (Addenbrooke's Hospital), and Cancer Research UK. The authors would like to thank those men with prostate cancer and the subjects who have donated their time and their samples to the Cambridge Biorepository, which were used in this research. We also would like to acknowledge to support of the research staff in S4 who so carefully curated the samples and the follow-up data (Jo Burge, Marie Corcoran, Anne George, and Sara Stearn)."

SEARCH
SEARCH is funded by a programme grant from Cancer Research UK [C490/A10124] and supported by the UK National Institute for Health Research Biomedical Research Centre at the University of Cambridge.

SFPCS
SFPCS was funded by California Cancer Research Fund grant 99-00527V-10182

STHM2
STHM2 was supported by grants from The Strategic Research Programme on Cancer (StratCan), Karolinska Institutet; the Linné Centre for Breast and Prostate Cancer (CRISP, number 70867901), Karolinska Institutet; The Swedish Research Council (number K2010-70X-20430-04-3) and The Swedish Cancer Society (numbers 11-0287 and 11-0624); Stiftelsen Johanna Hagstrand och Sigfrid Linnérs minne; Swedish Council for Working Life and Social Research (FAS), number 2012-0073
The authors acknowledge the Karolinska University Laboratory, Aleris Medilab, Unilabs and the Regional Prostate Cancer Registry for performing analyses and help to retrieve data. Carin Cavalli–Björkman and Britt-Marie Hune for their enthusiastic work as research nurses. Astrid Björklund for skilful data management. We wish to thank the BBMRI.se biobank facility at Karolinska Institutet for biobank services.
Sweeney_DFCI

SWOG-PCPT / SWOG-SELECT
PCPT/SELECT is funded by Public Health Service grants U10CA37429 and 5UM1CA182883 from the National Cancer Institute.
The authors thank the site investigators and staff and, most importantly, the participants from PCPT/SELECT who donated their time to this trial.

TAMPERE
The Tampere (Finland) study was supported by the Academy of Finland (251074), The Finnish Cancer Organisations, Sigrid Juselius Foundation, and the Competitive Research Funding of the Tampere University Hospital (X51003). The PSA screening samples were collected by the Finnish part of ERSPC (European Study of Screening for Prostate Cancer).
TAMPERE would like to thank Riina Liikanen, Liisa Maeaettaenen and Kirsi Talala for their work on samples and databases.

UKGPCS
UKGPCS would also like to thank the following for funding support: The Institute of Cancer Research and The Everyman Campaign, The Prostate Cancer Research Foundation, Prostate Research Campaign UK (now Prostate Cancer UK), The Orchid Cancer Appeal, The National Cancer Research Network UK, The National Cancer Research Institute (NCRI) UK. We are grateful for support of NIHR funding to the NIHR Biomedical Research Centre at The Institute of Cancer Research and The Royal Marsden NHS Foundation Trust. UKGPCS should also like to acknowledge the NCRN nurses, data managers and Consultants for their work in the UKGPCS.

UKGPCS would like to thank all urologists and other persons involved in the planning, coordination, and data collection of the study. KM and AL were in part supported from the NIHR Manchester Biomedical Research Centre

ULM
The Ulm group received funds from the German Cancer Aid (Deutsche Krebshilfe).

APCB
We also thank funding from the following: The APCB was jointly established by two enabling grants from the National Health and Medical Research Council (NHMRC) and Prostate Cancer Foundation of Australia (PCFA).

References

1. Lawrence, M.G., Veveris-Lowe, T.L., Whitbread, A.K., Nicol, D.L. & Clements, J.A. Epithelial-mesenchymal transition in prostate cancer and the potential role of kallikrein serine proteases. *Cells Tissues Organs* **185**, 111-5 (2007).
2. Barber, R.D., Harmer, D.W., Coleman, R.A. & Clark, B.J. GAPDH as a housekeeping gene: analysis of GAPDH mRNA expression in a panel of 72 human tissues. *Physiol Genomics* **21**, 389-95 (2005).

References

1. Lawrence, M.G., Veveris-Lowe, T.L., Whitbread, A.K., Nicol, D.L. & Clements, J.A. Epithelial-mesenchymal transition in prostate cancer and the potential role of kallikrein serine proteases. *Cells Tissues Organs* **185**, 111-5 (2007).
2. Barber, R.D., Harmer, D.W., Coleman, R.A. & Clark, B.J. GAPDH as a housekeeping gene: analysis of GAPDH mRNA expression in a panel of 72 human tissues. *Physiol Genomics* **21**, 389-95 (2005).
