# Supplementary material for: A PSA SNP associates with cellular function and clinical outcome in men with prostate cancer
Source: Nat Commun. 2024 Nov 6;15:9587. doi: 10.1038/s41467-024-52472-6 (PMC11541583; doi:10.1038/s41467-024-52472-6)
Supplement: Supplementary file 3 — Reporting Summary [file 41467_2024_52472_MOESM3_ESM.pdf]

nature portfolio

Corresponding author(s): Prof Jyotsna Batra

Last updated by author(s): Jul 26, 2024

Reporting Summary

Nature Portfolio wishes to improve the reproducibility of the work that we publish. This form provides structure for consistency and transparency in reporting. For further information on Nature Portfolio policies, see our Editorial Policies and the Editorial Policy Checklist.

Statistics

For all statistical analyses, confirm that the following items are present in the figure legend, table legend, main text, or Methods section.

n/a	Confirmed	
☐	☒	The exact sample size (n) for each experimental group/condition, given as a discrete number and unit of measurement
☐	☒	A statement on whether measurements were taken from distinct samples or whether the same sample was measured repeatedly
☐	☒	The statistical test(s) used AND whether they are one- or two-sided<br>*Only common tests should be described solely by name; describe more complex techniques in the Methods section.*
☐	☒	A description of all covariates tested
☐	☒	A description of any assumptions or corrections, such as tests of normality and adjustment for multiple comparisons
☐	☒	A full description of the statistical parameters including central tendency (e.g. means) or other basic estimates (e.g. regression coefficient) AND variation (e.g. standard deviation) or associated estimates of uncertainty (e.g. confidence intervals)
☐	☒	For null hypothesis testing, the test statistic (e.g. F, t, r) with confidence intervals, effect sizes, degrees of freedom and P value noted<br>*Give P values as exact values whenever suitable.*
☒	☐	For Bayesian analysis, information on the choice of priors and Markov chain Monte Carlo settings
☒	☐	For hierarchical and complex designs, identification of the appropriate level for tests and full reporting of outcomes
☒	☐	Estimates of effect sizes (e.g. Cohen's d, Pearson's r), indicating how they were calculated

Our web collection on statistics for biologists contains articles on many of the points above.

Software and code

Policy information about availability of computer code

Data collection	No computer code was used for this study.
Data analysis	Odds ratios (OR) and 95% confidence intervals (95% CI) were derived using SNPTEST (https://mathgen.stats.ox.ac.uk/genetics_software/snptest/snptest.html) or an in-house C++ program. Genotypes were called using Illumina's proprietary GenCall algorithm.

For manuscripts utilizing custom algorithms or software that are central to the research but not yet described in published literature, software must be made available to editors and reviewers. We strongly encourage code deposition in a community repository (e.g. GitHub). See the Nature Portfolio guidelines for submitting code & software for further information.

Data

Policy information about availability of data

All manuscripts must include a data availability statement. This statement should provide the following information, where applicable:

- Accession codes, unique identifiers, or web links for publicly available datasets
- A description of any restrictions on data availability
- For clinical datasets or third party data, please ensure that the statement adheres to our policy

Accession codes and publicly available datasets were not analysed or generated in this study. Source data are provided with this paper. Restrictions to data availability is included under the data availability statement. Further information and requests for resources and reagents should be directed to and will be fulfilled by the lead contact, Prof Jyotsna Batra (jyotsna.batra@qut.edu.au).

Research involving human participants, their data, or biological material

Policy information about studies with human participants or human data. See also policy information about sex, gender (identity/presentation), and sexual orientation and race, ethnicity and racism.

Reporting on sex and gender	Samples (Males) alone were included for analysis for this study and data (sex/gender) were collected by each cohort in accordance with Declaration of Helsinki. Samples genotypically identified as male (XY) alone were included in the analysis.
Reporting on race, ethnicity, or other socially relevant groupings	Cohorts provided core data on self-reported ethnic origin.
Population characteristics	Cohorts provided core data on disease status, age at diagnosis (observation or questionnaire for controls), family history, and clinical factors for cases (e.g. PSA at diagnosis, Gleason score, etc). For survival analysis follow-up on cause-specific death were also included.
Recruitment	Cohorts in the biobanking protocols described below were comprised of men and were recruited in accordance with Declaration of Helsinki and Research Ethics Committee guidelines described below.
Ethics oversight	Each study of PRACTICAL consortium was approved by each institutional review board (IRB) and informed consent was obtained from each participant. For MDC and VIP cohorts, the studies were approved by local institutional review boards (Research Ethics Board at Umeå University, for VIP and the Research Ethics Board at Lund University for MDC).

Note that full information on the approval of the study protocol must also be provided in the manuscript.

Field-specific reporting

Please select the one below that is the best fit for your research. If you are not sure, read the appropriate sections before making your selection.

☒ Life sciences ☐ Behavioural & social sciences ☐ Ecological, evolutionary & environmental sciences

For a reference copy of the document with all sections, see nature.com/documents/nr-reporting-summary-flat.pdf

Life sciences study design

All studies must disclose on these points even when the disclosure is negative.

Sample size	The cohort samples and size included were as a part of authors involvement in the international PRACTICAL consortium. Available samples were based on the availability of data to determine the association of the SNP with cancer risk and survival. Two independent cohorts were included as validation of our association analyses.
Data exclusions	For survival analysis, to include patients who died of other causes, prostate cancer-specific death samples were excluded.<br>For in vivo experiment, mice that died due to unrelated infections were excluded for data analysis.
Replication	All replications were successful.
Randomization	For in vivo experiments radomisation was followed where feasible: allocation of mice to different groups, injection for subcutaneous and intra cardiac models.
Blinding	For IHC intensity scoring analysis, the investigators were blinded to subject outcomes and sample origin.

Reporting for specific materials, systems and methods

We require information from authors about some types of materials, experimental systems and methods used in many studies. Here, indicate whether each material, system or method listed is relevant to your study. If you are not sure if a list item applies to your research, read the appropriate section before selecting a response.

Materials & experimental systems

n/a	Involved in the study
☐	☒ Antibodies
☐	☒ Eukaryotic cell lines
☒	☐ Palaeontology and archaeology
☐	☒ Animals and other organisms
☒	☐ Clinical data
☒	☐ Dual use research of concern
☒	☐ Plants

Methods

n/a	Involved in the study
☒	☐ ChIP-seq
☐	☒ Flow cytometry
☒	☐ MRI-based neuroimaging

Antibodies

Antibodies used	Primary anti-PSA antibody (Dako, #A0562).
Validation	This anti-PSA antibody was used routinely used in our lab for IHC analysis. A dilution of 1:5000 was utilised for IHC staining of FFPE sections in this study. There are 89 publications at the manufacturer's site for this antibody attesting to the validity and staining specificity.

Eukaryotic cell lines

Policy information about cell lines and Sex and Gender in Research

Cell line source(s)	PC-3, LNCaP cells. Human (male) prostate cancer. MSK3, a mucinous adenocarcinoma cell line isolated from retroperitoneal lymph node were generated at Memorial Sloan Kettering Cancer Center as previously described (Gao, D., Vela, I., et al., Cell 2016). All patients provided informed consent and samples were acquired under MSKCC IRB-approved protocols # 06-107 and 12-001. Endothelial HUVEC cell lines (used to test PSA variants containing PC-3 cell line conditioned media) were sourced from American Type Culture Collection (ATCC). HUVECs (used to test recombinant PSA protein) were isolated from umbilical veins at University of Helsinki, Finland.
Authentication	All cell lines were authenticated by STR profiling.
Mycoplasma contamination	Cell lines were routinely monitored for mycoplasma contamination and were tested negative.
Commonly misidentified lines (See ICLAC register)	No misidentified lines were used in the study.

Animals and other research organisms

Policy information about studies involving animals; ARRIVE guidelines recommended for reporting animal research, and Sex and Gender in Research

Laboratory animals	5-6 week old male NOD SCID gamma (NSG) mice were utilised.
Wild animals	No wild animals were used in the study.
Reporting on sex	Male animals were used for studying prostate cancer.
Field-collected samples	n/a
Ethics oversight	In-vivo studies were performed in accordance with guidelines of the Animal Ethics Committees of The University of Queensland (AEC number: 091/17) and Queensland University of Technology.

Note that full information on the approval of the study protocol must also be provided in the manuscript.

Plants

Seed stocks: n/a

Novel plant genotypes: n/a

Authentication: n/a

Flow Cytometry

Plots

Confirm that:

☒ The axis labels state the marker and fluorochrome used (e.g. CD4-FITC).

☒ The axis scales are clearly visible. Include numbers along axes only for bottom left plot of group (a 'group' is an analysis of identical markers).

☒ All plots are contour plots with outliers or pseudocolor plots.

☒ A numerical value for number of cells or percentage (with statistics) is provided.

Methodology

Sample preparation: PC-3 and LNCaP cells were trypsinised and resuspended in 1 mL basic sort buffer (containing 2% FBS and 2mM EDTA) and filtered through the strainer for flow cytometry analysis.

Instrument: Astrios cell sorter (Beckman Coulter, Australia)

Software: FlowJo

Cell population abundance: Initial cell populations were selected through the forward and side scatter plots by excluding debris and dead cells (smallFSC and SSC) followed by FSC-A/FSC-H gating to select singlet cells.

Gating strategy: All samples were FSC-A and SSC-A gated, followed by FSC-A/FSC-H gating to select singlet cells. Uninfected control cells were used to set gates.

☒ Tick this box to confirm that a figure exemplifying the gating strategy is provided in the Supplementary Information.
